# The recent trend in mycobacterial strain diversity among extra pulmonary lymph node tuberculosis and their association with drug resistance and the host immunological response in South India

**DOI:** 10.1186/s12879-020-05597-0

**Published:** 2020-11-26

**Authors:** Shanmugam Sivakumar, Yuvaraj Chandramohan, Gokul Raj Kathamuthu, Gomathi Sekar, Devika Kandhasamy, Venkatesan Padmanaban, Syed Hissar, Srikanth P. Tripathy, Ramalingam Bethunaickan, Baskaran Dhanaraj, Subash Babu, Uma Devi Ranganathan

**Affiliations:** 1 Department of Bacteriology, National Institute for Research in Tuberculosis, Chetpet, Chennai, 600 031 India; 2 Department of Immunology, National Institute for Research in Tuberculosis, Chetpet, Chennai, 600 031 India; 3 National Institute of Health -International Center for Excellence in Research - National Institute for Research in Tuberculosis, Chennai, India; 4 Department of Clinical Health Research, National Institute for Research in Tuberculosis, Chetpet, Chennai, 600 031 India; 5 National Institute for Research in Tuberculosis, Chetpet, Chennai, 600 031 India

**Keywords:** Extra pulmonary tuberculosis, *Mycobacterium tuberculosis*, Spoligotyping, Th1 and Th17 cytokines, Lymph node, Mycobacterial lineage

## Abstract

**Background:**

Tuberculosis (TB) though primarily affects the lungs it may also affect the other parts of the body and referred as extra pulmonary (EPTB). This study is focused on understanding the genetic diversity and molecular epidemiology of *Mycobacterium tuberculosis* (*M.tb*) among tuberculous lymphadenitis (TBL), a form of EPTB patients identified in Chennai, Tamil Nadu.

**Methods:**

The genetic diversity was identified by performing spoligotyping on the *M.tb* clinical isolates that were recovered from lymph node samples. A total of 71 *M.tb* isolates were recovered from extra pulmonary lymph node samples and subjected to Drug susceptibility testing and spoligotyping was carried out. In addition, immunological characterization from blood of same individuals from whom *M.tb* was isolated was carried out between the two major lineages groups East African Indian 3 (EAI3) and non-EAI3 strains by ELISA. The results of spoligotyping patterns were compared with the world Spoligotyping Database of Institute Pasteur de Guadeloupe (SpolDB4).

**Results:**

We found 41 spoligotype patterns and their associated lineages. Out of 41 spoligotype pattern, only 22 patterns are available in the spoldB4 database with Spoligotype international Type (SIT) number and remaining patterns were orphan strains without SIT number. The most predominant spoligotype lineage that was found in lymph node sample in this region of India was EAI (36), followed by central Asian strain (CAS) (6), T1 (5), Beijing (3), Latin American & Mediterranean (LAM) (2), U (1), X2 (1) and orphan (22). In addition to EAI, CAS and Beijing, our study identified the presence of orphan and unique spoligotyping patterns in Chennai region. We observed six drug resistant isolates. Out of six drug resistant isolates, four were resistant to isoniazid drug and associated with EAI family. Moreover, we observed increased levels of type 2 and type 17 cytokine profiles between EAI3 and non-EAI family, infected individuals.

**Conclusions:**

The study confirms that EAI lineage to be the most predominant lineages in EPTB patients with lymphadenitis and were found to have increased type 1 and type 17 proinflammatory cytokine profiles.

**Supplementary Information:**

The online version contains supplementary material available at 10.1186/s12879-020-05597-0.

## Background

Tuberculosis (TB) occurs worldwide and it is caused by an infectious agent *Mycobacterium tuberculosis* (*M.tb*). According to World health organization (WHO), there were 1.3 million TB deaths was reported among HIV negative people and an additional 3, 00,000 deaths among HIV-positive people were reported in 2017. TB affected about 10 million people; among them 90% were adults, 9% were people living with HIV [1]. The two types of clinical manifestations of TB are pulmonary (PTB) and Extra-pulmonary (EPTB) tuberculosis. EPTB involves TB affects body parts other than the lungs, which include lymph nodes, skins, joints, or meninges. Among 6.4 million incident cases, 14% represents extrapulmonary TB [1]. In India, about 15 to 20% of EPTB occurs in immune-competent patients and 50% of cases were reported in HIV-positive individuals [2]. Among them, lymph node TB (35%), pleural TB (20%), bone marrow TB (10%), genitourinary TB (9%), cerebrospinal, abdominal, skin, etc. accounts for 26% cases [2]. Though EPTB is not contagious, it has a huge impact on mortality and morbidity rate worldwide. *Mycobacterium tuberculosis* complex (MTBC) is the most common etiological agent in TB including EPTB and their diagnosis and therapeutics are highly challengeable [3].

Epidemiology of EPTB can be analyzed by genotyping of *Mycobacterium tuberculosis*, which will be helpful to detect unsuspected transmission, reinfection and relapse and to identify false-positive cultures [4]. The available genotyping methods are IS6110-RFLP, Polymorphic GC-rich repetitive sequences (PGRS)-RFLP, Mycobacterial interspersed repetitive unit (MIRU)-VNTR typing and Spoligotyping. Of the available genotyping tools, Spoligotyping and MIRU-VNTR is considered the most suitable tool in clinical settings [5].

Since Spoligotyping (Spacer Oligonucleotide typing) can simultaneous detect and type different lineages of *M. tuberculosis* bacterial complex (MTBC). The clinical data obtained by this method had a significant impact on immune surveillance and drug treatment regimens [6]. Previous studies demonstrated that *M. tuberculosis* strains have different epidemiology patterns. Evidence from a variety of sources suggests that MTBC lineages have different phenotypic properties. According to Centers for Disease Control and Prevention (CDC), MTBC genetic lineages are of two categories: Modern lineages and Ancestral lineages. The Modern lineages include East-Asian (Beijing), East-African-Indian (EAI) and Euro-American. The Ancestral lineages include Indo-Oceanic, *M. bovis* and *M. africanum* (West African 1 & West African 2) [7]. Therefore, spoligotypic information on clinical isolates can reveal the phylogenetic and evolutionary relationships among those strains. Previous studies have also documented that EAI (ancient strain) trigger significantly increased expression of certain (TNF-α, IL-1β, IL-12) cytokines and apoptosis than Beijing (modern lineage) and H37Rv (laboratory strain) up on in ex-vivo. Also, it has been shown that ancient strains were less lethal when compared to modern strains and they stimulate greater pro-inflammatory host response. In contrast, infection with Mtb Beijing strains might induce non-uniform immune responses [8–11]. In a given situation, characterization of those lineages in EPTB may provide better insights on strain pathogenicity and further will help us to document global tuberculosis epidemiology [12]. Therefore, in this study, we aimed to genotype 71 clinical isolates of *M. tuberculosis* from patients with extrapulmonary tuberculosis (EPTB) to identify the predominant spoligotyping pattern of strains in lymph node samples isolated from Southern India and studied strain specific immune responses among the infected individuals.

## Methods

### Clinical setting

The strains were received from an ongoing, clinical trial to study ‘the efficacy and tolerability of a 4-month regimen containing ofloxacin compared to the standard 6-month regimen in the treatment of patients with superficial lymph node TB by National Institute for Research in Tuberculosis (NIRT), Chennai which is a Supranational Reference Laboratory and a WHO collaborating center for TB research and training. From this ongoing study, 71 mycobacterial isolates retrieved from suspected extra pulmonary cases and utilized for performing mycobacterial strain characterization (from the year 2013–2015). The Institutional Ethical Committee (IEC) of National Institute for Research in Tuberculosis (NIRT) approved the study and written informed consent was obtained from the patients before collecting the samples (NIRT IEC 2009 007).

### Sample processing, biochemical characterization and identification

The lymph node samples were collected and transported in Kirchner’s medium to the laboratory. Lymph node sample processing: The biopsy sample (Lymph node) is cut into small pieces and grinded with sterile Teflon grinding rod, direct smear is made from the homogenate. The homogenate is treated with 1 ml 5% H_2_SO_4_ for 15 min and neutralized and with sterile distilled water. The deposit is washed and inoculated onto two slopes each of plain Lowenstein Jensen (LJ) and LJ Sodium Pyruvate solid medium, remaining deposit is inoculated into two bottles of SK. LJ medium and SP medium are read for 8 weeks every week before declaring as negative. The SK medium is subculture on to LJ medium if positive. The drug susceptibility testing for First- and second-line anti-TB drugs were carried out for 71 isolates of *M.tb*. The minimum inhibitory concentration (MIC) was used to predict the sensitive and resistance patterns as per the guidelines recommended by WHO for First- and second-line anti-TB drugs (https://www.who.int/tb/publications/2018/WHO_technical_report_concentrations_TB_drug_susceptibility/en/). In Brief MIC method was performed using 4 mg moist weight per microlitre (ml) of culture suspension, one-third loopful of 2–3 wk. old culture on L-J medium was suspended in 1 ml of sterile distilled water and vortexed to obtain uniform suspension. The coarse particles or clumps in the suspension were allowed to settle at room temperature. For MIC method, 10 μl of the suspension was inoculated onto drug containing and drug free L-J medium. Results were read after 28 days of incubation at 37 °C for MIC methods. Isolates with ≥20 colony counts (1+ grading) were considered resistant to the particular drug concentration. In addition, MPT-64 Ag detection test were performed for these 71 isolates using SD Bioline TBAg MPT64 Rapid (Standard diagnostics Seoul, South Korea) Kit [13] to confirm *M.tb*.

### DNA extraction

The DNA extraction from the confirmed *M. tuberculosis* positive patients was carried out as described [14]. The mycobacterial cultures were grown in LJ medium and scrapped in to 1 ml TE (Tris EDTA) buffer. At 80 °C, the cultures were heat killed for 30 min. To that, 50 μl of lysozyme (10 mg/ml) was added and the isolates were incubated at 37 °C. On the next day, 70 μl of 10% SDS and 6 μl of proteinase K (50 mg/ml) was added and further incubated at 65 °C for 15 min. Then, 100 μl of 5 M NaCl and 80 μl of CTAB were added, incubated again at 65 °C for 15 min. To the culture, 700 μl of chloroform/isoamyl alcohol (24:1) was added and centrifugation was carried out for 20 min at 10,000 g at 25 °C. The supernatant was collected into a new Eppendorf tube. To precipitate the DNA, 600 μl of ice-cold iso-propanol was added to the tubes and incubated at − 20 °C overnight. On the third day, Eppendorf tubes were subjected to centrifugation at 12,000 rpm for 15 min and the pellet was washed with 500 μl of 70% ethanol. The extracted genomic DNA was re-suspended in TE buffer and stored at 20 °C until use. To confirm the presence of DNA, the extracted DNA was run on 1% agarose gel electrophoresis and quantified using Nano drop.

### Spoligotyping

Spoligotyping was performed as described previously [15]. DRa and DRb primers were used for amplifying the direct repeat (DR) region in the genome of MTB complex. *M. tuberculosis* H37Rv strain and *Mycobacterium bovis* BCG P3 chromosomal DNA were used as positive controls. The molecular biology grade water served as negative control. The commercial membrane pre-coated with spacer-oligos, which represents the spacer region of known sequences, was used for hybridizing the amplified product. The membrane was incubated with streptavidin-peroxide and ECL to visualize the presence/absence of spacer on X-ray film as black squares. Based on the combination of the presence/absence of theses spacers in a M.tb strain is distinguished as spoligotype. The available spoligotyping patterns in international spoligotype database (SpoIDB4) were used for comparing the spoligotyping patterns of lymph node samples obtained from this study and SIT number is assigned.

### Elisa

In order to study the host immune response, plasma samples obtained from EPTB infected with respective strains were used to measure the systemic levels of different (IFNγ, TNFα, IL-2, IL-4, IL-5, IL-13, IL-12, IL-17A, IL-22, IL-10, IL-1α, IL-1β, IL-6 and GM-CSF) cytokines and CC/CXC chemokines (CCL-1/I-309, CCL2/MCP-1, CCL3/MIP1α, CCL4/MIP-1β and CCL11/eotaxin, CXCL-1/Gro-α, CXCL2/ Gro-β, CXCL9/MIG, CXCL10/IP-10 and CXCL11/ITAC 1) by bio-plex ELISA. The experiment was carried out according to the manufactures instruction provided (R & D systems).

### Data analysis

The immunological parameters were analyzed using GraphPad PRISM (GraphPad Software, Inc.,San Diego, CA, USA). Geometric means (GM) were used for measurements of central tendency. Finally, Mann-Whitney U test was used to find the statistically significant differences among the cytokines and chemokines.

## Results

We recovered 71 *Mycobacterium tuberculosis* strains from lymph node collected from EPTB patients recruited under this study. Majority of the patients recruited in this study were aged more than 18 years and other demographic and clinical details were reported in supplementary Table 1.

### Drug resistance and EPTB recurrence

Out of the 71 isolates tested, 65 isolates were sensitive to all first line and second line anti-TB drugs. Four isolates were resistant to isoniazid; one isolate was mono resistant to rifampicin and one isolate shows resistant to ofloxacin (Table 1).

**Table 1:** Shows the number of different lineages identified by SpoldB4 database

S. NO	SPOLIGOTYPES	NO OF ISOLATES (NO OF RESISTANT STRAINS)
1.	Beijing 1	3 (c-1)
2.	CAS 1345	1
3.	CAS 142	1
4.	CAS 601	1
5.	CAS1_DELHI 26	3
6.	EAI 3 Orphan	9 (a-3)
7.	EAI 5 Orphan	9
8.	EAI1_SOM 270	1
9.	EAI1_SOM 48	3 (d-2)
10.	EAI1_SOM 734	1
11.	EAI3_IND 11	20
12.	EAI3_IND 473	1
13.	EAI5 126	2
14.	EAI5 340	1
15.	EAI5 355	1
16.	EAI5 474	1
17.	EAI5 517	1
18.	EAI5 733	1
19.	EAI5 938	2
20.	EAI6_BGD1 591	1 (a-1)
21.	Harleem1 Orphan	3
22.	LAM9 42	2
23.	Orphan	22
24.	S Orphan	1
25.	T1 53	5 (b-1)
26.	U 172	1
27.	X2 137	1

a-Isoniazid resistant; b-Rifampicin resistant; c-Ofloxacin resistant; d-Recurrence

### Spoligotyping

**Fig. 1:**
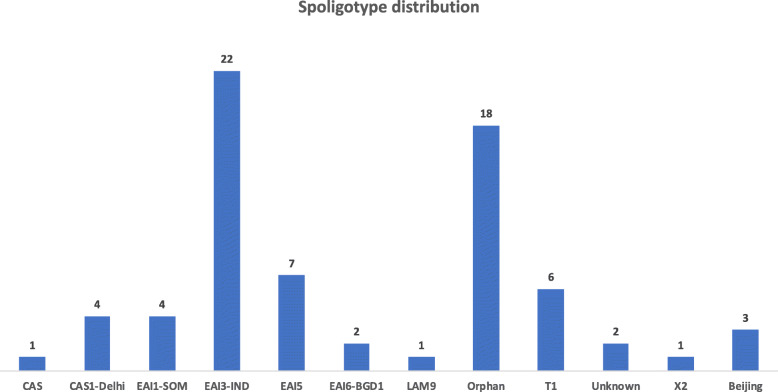
Spoligotype patterns and their associated lineages of 71 isolates from lymph node samples

**Fig. 2:**
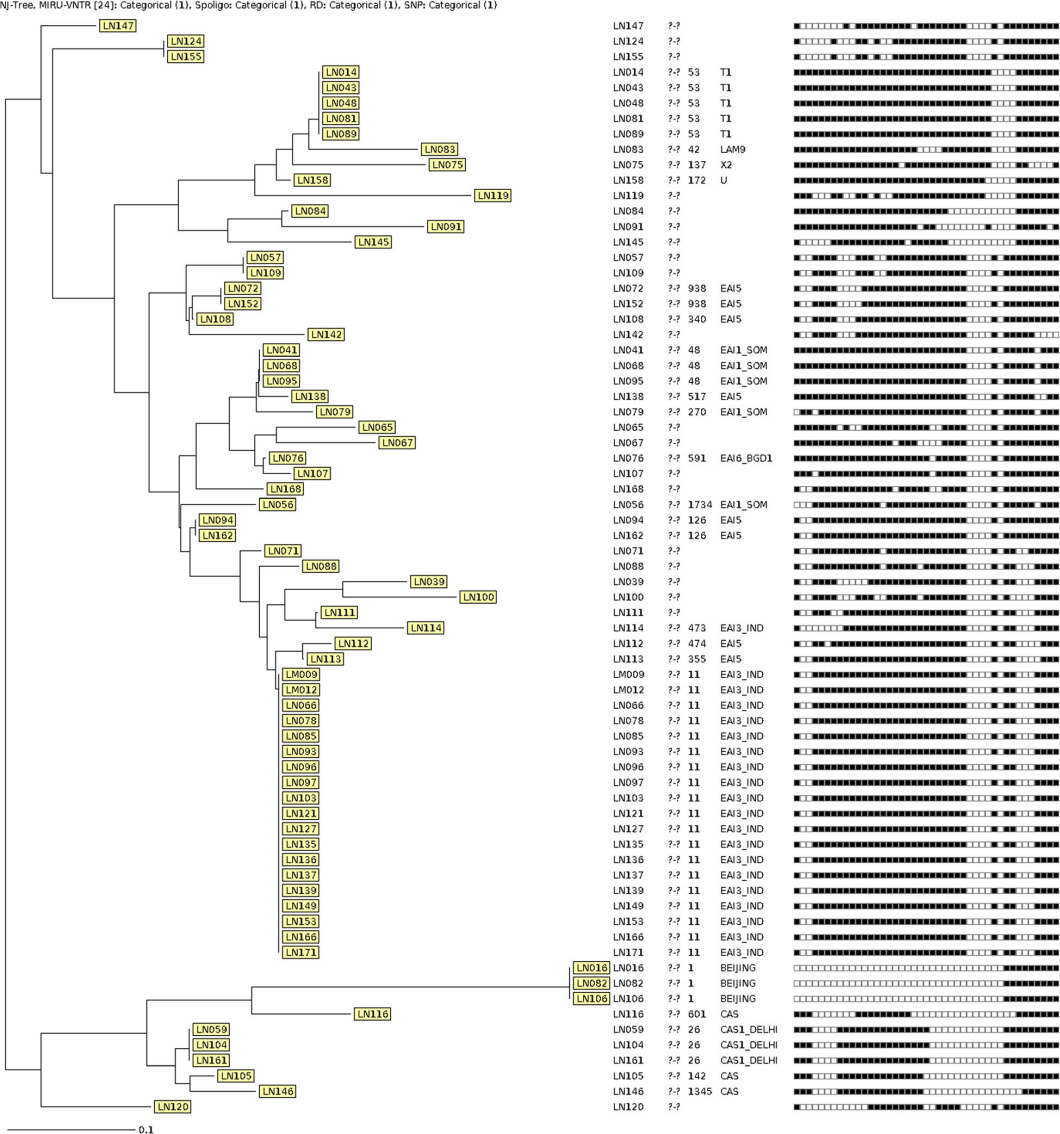
Phylogenetic tree of 71 isolates from lymph node samples

The isolated cultures from the lymph node were subjected to spoligotyping and it resulted in 41 spoligotype patterns as per the SpolDB4 database [16]. The resulted 41-spoligotype patterns and their associated lineages are shown in Fig. 1. The phylogenetic tree for the spoligotype pattern was generated using unweighted pair group method and arithmetic mean (Fig. 2). Out of 41 spoligotype pattern, only 22 patterns are available in the spoldB4 database with Spoligo international Type (SIT) number and remaining patterns were identified as orphan strains without SIT number. The most predominant spoligotype lineage that was found in lymph node sample in this region of India was EAI (36), followed by CAS (6), T1 (5), Beijing (3), LAM (2), U (1), X2 (1) and orphan (22). We looked at the age and gender association, though the numbers (*n* = 71) are few to draw any conclusion, we noticed females were more (74.6%) represented compared to males (25.4%). Majority of the common strains are EAI3_IND and Orphans ND and strains are represented by all age group (Supplementary Fig. 2A & B).

### Sub-lineages identified from lymph node samples

Among the EAI family, the most predominant lineage was EAI3 (21 Nos) followed by EA15 (9 Nos), EAI1 (5 Nos) and EAI6 (1 Nos). The sub-lineages identified from lymph node samples are summarized in Table 1. A total of 21 isolates belongs to EAI3, comprising 20 isolates in EAI3_IND and 1 isolate in EAI3_IND 473. The next predominant lineage of EAI family is EAI5 (9 No) which are further classified in to different sub lineages as follows: EAI5 126 (2 Nos), EAI5 340 (1 No), EAI5 355 (1 No), EAI5 474 (1 No), EAI5 517 (1 No), EAI5 733 (1 No) AND EAI5 938 (2 Nos). The third predominant lineage of EAI family is EAI1 (5 Nos) and was further identified to belong to four sub-lineages EAI1_SOM 270 (1 No), EAI1_SOM 48 (3 Nos) andEAI1_SOM 734 (1 No) EAI6 (1 No). Total of 18 new variants of EAI were found without SIT numbers. The next prevalent lineage among 71 isolates of lymph node sample is CAS family (6 Nos). A total of 6 isolates belongs to CAS, comprising 3 isolates in CAS_DELHI 26 followed by CAS 1345 (1 No), CAS 142 (1 No) and CAS 601 (1 No). The existing spoldB4 database was compared and the patterns, which do not match the existing information, are called orphan strains. A total of 22 orphan strains were identified from this study.

### Spoligotyping pattern and their associated drug resistance

Out of 71 isolates tested, 65 were sensitive to all the first- and second-line anti-TB drugs and belong to different lineages like EAI, CAS, LAM, U and X2. The spoligotype patterns and their associated drug resistance were analyzed in this study. We observed six drug resistant isolates. Out of six drug resistant isolates, four were resistant to isoniazid drug and associated with EAI family. Mostly, the sub-lineages of EAI3 orphan (3 Nos) and EAI6_BGD1 (1 No) are associated with H mono resistance (Table 1). The remaining two strains were mono resistant to rifampicin and ofloxacin and belong to T1 and Beijing lineage respectively. In addition, we found recurrence of the disease in two patients out of 71 patients tested, they were primarily infected with EAI1_ SOM sub-lineage, which is different from the most prevalent circulating EAI3 strains in south India (Table 1).

### Differential immune response among patients infected with EAI3 and non – EAI 3 strains

**Fig. 3:**
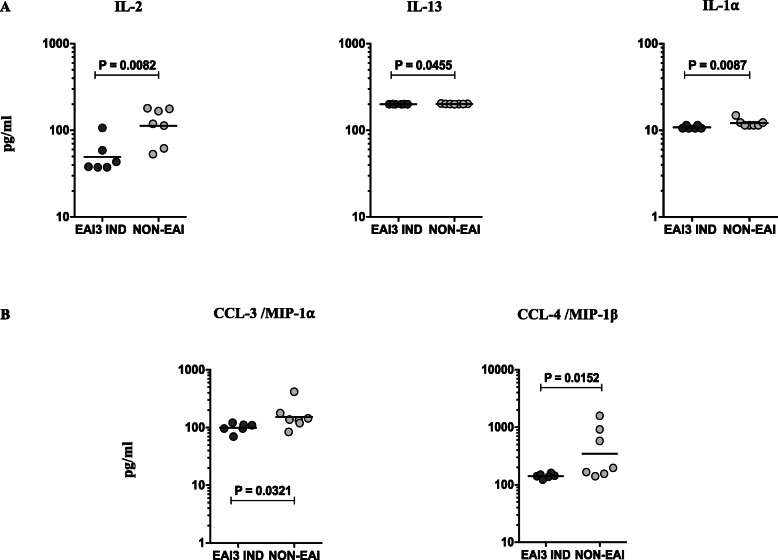
Altered systemic levels of cytokine and decreased chemokines between the EAI3 IND and non-EAI individuals. **a** The systemic levels of different cytokines such as IL-2, IL-13, IL-1α and (**b**) the plasma levels of chemokines (CCL3/MIP-1⍺ and CCL4/MIP-1β) were measured by ELISA between the EAI3 IND (*n* = 6) and NON-EAI (*n* = 7) strain infected individuals. The data were shown as scatter plots with each circle represents a single individual and the bar represents the geometric mean. *P* values were calculated using the Mann-Whitney U test

To determine whether any significant immunological differences are associated with the individuals infected with either EAI3 India or non-EAI3 strains, we have measured the different (type 1, type 2, type 17, IL-1 family and pro-inflammatory) cytokines using the available plasma samples of respective EPTB individuals (Supplementary Fig. 1). Among them, the plasma levels of IL-2 and IL-1α were significantly higher in individuals infected with non-EAI3 India strains upon comparison with EAI3 India strains. In contrast, IL-13 cytokine level was significantly reduced in non-EAI3 India strain infected individuals when compared to EAI3 India strains (Fig. 3a). Similar to cytokines, we have also examined the systemic levels of different CC chemokines between the EAI3 India and non-EAI strains. As shown in Fig. 3b, the CC (CCL-3/ MIP-1⍺ and CCL-4/ MIP-1β) chemokines were significantly different (diminished in EAI3 India strain infected individuals) between the two study groups.

## Discussion

A major public health concern, which still remains and contributes to the world epidemiology, is tuberculosis. For development of effective infection control and vaccines, the frequency of various *M.tb* genotype and their transmission patterns need to be analyzed using molecular tools such as spoligotyping and MIRU-VNTR. To differentiate recurrence, re-infection and mixed infections in multiple strains of *M.tb* these genotyping methods are widely used [17, 18]. Previous reports from our group have already reported the circulating lineages of south India from pulmonary TB samples [19]. However, this was the first report from our group, which focuses on circulating lineage of extra pulmonary lymph node samples from ongoing clinical trials. In this study, we mainly focus on circulating lineages of extrapulmonary lymph node samples and their associated drug resistance pattern. Not many molecular epidemiological studies have been performed for lymph node extra pulmonary sample. In this context, this study provides a great insight and a better understanding of circulating lineages and their associated drug resistance patterns of extra pulmonary lymph node samples.

We performed Spoligotyping and Immunological assays for the extrapulmonary lymph node samples to understand the genetic diversity of different circulating strains that were recovered from ongoing clinical trial. Our present study identified 76.05% of EAI strains from Chennai, which was isolated from lymph nodes samples, and found to be most predominant strains circulating in south India. This is in line with the previous studies, which identified 67.5 and 59.3% of EAI strains from pulmonary *M.tb* isolates [19, 20]. In comparison with North India, the high prevalence of EAI strains in pulmonary MTB isolates has already been reported in Kerala and Chennai [19–22]. The highest percentage of EAI strains (89%) were previously reported in Kerala [20]. Similarly, Joseph and co-workers also reported 64.3% of EAI strains in 2013 [21]. Various reports from North India mentioned 3.8% EAI strains from Delhi [18], 4.44% EAI strains from Maharashtra [23], 9.6% in UP and Varanasi [24] and 10% from the whole of north India [3]. Singh et al., 2007 reported that EAI lineages are more predominant in south India, whereas the CAS family is predominating the northern parts of India [25]. The origin, evolution and migration of two populations in India and the differences in lineages shows the presumption that *Mycobacterium tuberculosis* strains might have originated and evolved in a different way [3]. Similar study showed that Euro-American lineage were three times more likely to generate a secondary case during the 11-year study period than patients infected with any other strain. The data clearly shows that the immune response and genetic background plays a major role in deciding the infection with a particular lineage of *Mycobacterium tuberculosis* [26]. In the present study, we identified one EAI6_BGD1 from lymph node samples. A similar finding has been reported for the first time in Puducherry [27]. In the current study, drug susceptibility testing was carried out for all the isolates of lymph node samples. Majority of the strains were susceptible to first line drugs and six isolates were resistant to drugs like rifampicin, isoniazid and ofloxacin. Out of six isolates tested, all the four isolates are from EAI family and were resistant to Isoniazid. These resistant strains belong to EAI3 orphan strains (3 Isolates) and EAI6_BGD1 (1 Isolate).

The central and Middle Eastern Asia (CAS) family were the second largest Clade identified in our study; this is reported to be the predominant lineage in North India previously. Our study identifies 7.89% of CAS strain from lymph node samples, which accounts for the low prevalence of CAS family in Chennai. This is similar to the findings of previous research workers who also recorded the low prevalence of CAS family in south India, which accounts 1.5% in Trivandrum [20], 3.5% in Chennai [19] and 3.5% in Kerala [21]. Contrary to this low prevalence of CAS family in south India, North India recorded a very high prevalence of CAS family (57.2%) from extra pulmonary samples [3]. Similarly, Singh et al., 2007 reported 56.5%, 53.6 and 44.6% from Delhi, Pune and Lucknow, respectively [20]. This evidence clearly suggests that there are contrasting lineages circulating in north and south India. It is well-established historical fact that North Indians belongs to the Aryan descent whereas, South Indians belongs to Dravidian culture. This might be contributing for the demarcation of prevalent strains in different parts of India. In contrast, a study by Thomas et al., reported the higher prevalence of CAS family in Andhra Pradesh. This could be due to the migration of people to Hyderabad from Middle East countries and north India which contributes for the increased prevalence of CAS family.

The prevalence for Beijing strains in different parts of India ranges from 2.2% [19] to 11.5% [20] for pulmonary specimens. In the case of extra pulmonary specimens, the prevalence was slightly higher with the percentage of 10 and 18.8% [3, 28]. The Beijing strain was found to be more prevalent in multi drug extrapulmonary TB specimen and treatment failure cases with the percentage of 63.6% [23, 28]. In our study, Beijing strain was identified at a rate of 3.94% from lymph node. One of our lymph node samples were found to be resistant to Ofloxacin and associated with Beijing family. Previous studies from rural areas of Hyderabad and Mumbai have shown that Beijing strains were absent. However, in urban population of Andhra Pradesh it was prevalent at a rate of 6.8 and 10.4% among treatment failure cases [23, 29]. In addition to Beijing, the high rate of drug resistance is also associated with Haarlem lineages. This study reports the prevalence of Haarlem strain at a rate of 3.94% but none of these lineages contributed for the drug resistance genotypes. Interestingly, outside India the frequency of Beijing strains was more (56%) in TB meningitis patients which was reported by two consecutive studies from Bangkok and Thailand [30, 31].

In our study among the orphans, we found 22 unique patterns which were missing in SpolDB4, 9 *M.tb* Strains show features of EAI3 strains and 9 show similarity to EAI5 and three to Haarlem and one to S spoligotype. Previous reports by Desikan and his colleagues from Madhya Pradesh reported three EPTB isolates were found to be orphan [32]. In addition to this we also found very rare genotypes like LAM (2.63%), X2 (1.31%), U (1.31%) and S (1.31%) lineages in our study. This is in line with a study by Sankar et al. [3] who reports the rare isolates of LAM and X2 from extra pulmonary specimens. Furthermore, our study also identified Five T1 isolates from extra pulmonary specimens and for the first time one such report is also reported by Kandhakumari et al. [27] in India. In addition, drug susceptibility testing also found among six isolates tested; only one isolates belonging to the T1 lineage was found resistant to one of the first line drug rifampicin. We had four relapse in our study and genotyping results was available for two of them and both belong to EAI1_SOM. Recurrent infections have been reported earlier by us in HIV infected TB patients by EAI1_SOM *M.tb* strains [33]. Further studies need to carry out to explore association of EAI1_SOM *M.tb* strains in recurrence.

Furthermore, exogenous reinfections were observed by many researchers in both immunocompetent and immunosuppressed individuals. Mostly, drug resistant strains and exogenous reinfections from drug sensitive organisms have been reported in so many lineages. Two patients in our study had a recurrent TB infection. Moreover, immunological characterization studies were performed between EAI and Non-EAI family in order to understand the differences in cytokine profiles which confers drug resistance to this particular lineage. Immune protection against tuberculosis are mediated by the induction of T cell responses specifically through type 1 and type 17 cytokines [34]. Besides, IL-1 family (IL-1α, IL-1β and IL-18) and IL-12 cytokine are also necessary for protection against Mtb [35]. However, the relationship or the association of strain specific immune mediated responses in EPTB individuals has not been examined so far. Hence, for the first time we show there was significant immunological differences were observed between the individuals infected with EAI3 India and non-EAI strains. The major differences observed are for IL-2, IL-1α (reduced in EAI3 India strain) and IL-13 (increased in EAI3 strain). Decreased IL-2 levels in the EAI3 India strain infected individuals suggest that the former is associated with loss of immune protection and persistent chronic infection associated in EPTB individuals. The complete loss of IL-2 eventually leads to reduced immunity and expansion of Mtb growth and increased IL-2 provides protection along with substantial expansion of central memory T-cells [36, 37]. Like IL-2, IL-1 family cytokines are also equally important for immune protection and our data shows diminished levels of IL-α in EAI3 India strain infected individuals than the non-EAI. Previous studies also shown that deficiency in the IL-1 family cytokines are more susceptible to TB infection [35]. Finally, Type 2 cytokine (IL-13) were significantly enhanced in individuals infected with EAI3 India strain. Type 2 cytokines were harmful to host immune response to TB disease [34] and increased levels associated with chronic infection among the EAI3 India strain than non-EAI strain infected individuals. Therefore, individuals with EAI3 infected strains might have suppressed host protective (Type 1) responses mediated by Type 2 cytokines. Apart from those, type 1 (IFNγ, TNFα), type 2 (IL-4, IL-5), type 17 (IL-17, IL-22), regulatory (IL-10) and pro-inflammatory (IL-6, IL-12 and GM-CSF) cytokines were not significantly different between the two strains (data were not shown).

In addition, we have also analyzed some of the major CC and CXC (CCL-1/I-309, CCL2/MCP-1, CCL3/MIP1α, CCL4/MIP-1β and CCL11/eotaxin, CXCL-1/Gro-α, CXCL2/ Gro-β, CXCL9/MIG, CXCL10/IP-10 and CXCL11/ITAC 1) chemokines between the EAI3 India and non-EAI strain infected individuals. Interestingly we found CCL3/MIP1α and CCL4/MIP-1β were higher in non-EAI strain infected individuals (Supplementary Fig. 1). Both chemokines have an important role in migration, homing, recruitment and stimulation of leukocytes to the sites of inflammation [38]. Hence the immune system fails to protect individuals infected with EAI3 strains against EPTB infection. Further, we have also examined these cytokine and chemokine response to in drug sensitive (DS) versus Ofloxacin mono resistant (OMR) strains. Similar results were also observed between the two groups where increased Type 2 (IL-4 and IL-13) and Type 17 (IL-17 and IL-22) cytokines were observed in OMR strain infected individuals (Supplementary Fig. 1). It has been shown that Type 17 cytokine plays a crucial function in controlling Mtb through recruitment of immune cells, effective granuloma formation at early stages of infection. Meanwhile increased production might have the detrimental effects by promoting immunopathology [39]. Similar to EAI3 and non-EAI3 groups, the other cytokines and chemokines do not possess any significant differences between the DS and OMR (data were not shown) strains infected individuals. These observations reinforce that cytokines or chemokines involved in the host protection are compromised or suppressed in the EAI3 India strain or in OMR resistant strain infected individuals. Using only spoligotyping as the method for molecular epidemiology was limitation of our study, but we wanted to classify our strains into broader range, so that we can better study the immune response developed by the different spoligotypes. The other limitation is small sample number and drug resistance strains in EPTB samples analyzed.

## Conclusion

The present study provides molecular epidemiological data of mycobacteria in lymph node samples isolated from extra-pulmonary patients from south India. The study confirms that EAI lineage to be the most predominant lineages in EPTB patients with lymphadenitis and were found to have increased type 1 and type 17 proinflammatory cytokine profiles. The presence of orphan and unique spoligotyping patterns in this study identifies the genetic diversity of *M.tb* strains in Chennai region and more molecular epidemiological studies are recommended in lymph node samples throughout India to elucidate their role in TB lymphadenitis.

## Supplementary information

Additional file 1: **Supplementary Table 1.** Demographic and clinical details of the study population

Additional file 2: **Supplementary Fig. 1.** Diminished plasma levels type 2 and type 17 cytokines associated with individuals infected with drug sensitive strain. The systemic levels of IL-4, IL-13, IL-17A and IL-22 were measured by ELISA between the drug sensitive (DS, *n* = 10) and orphan mono resistant (OMR, *n* = 3) strain infected individuals. The data were shown as bar graph and *P* values were calculated using the Mann-Whitney U test.

Additional file 3: **Supplementary fig. 2A.** The M.tb spoligotypes distribution among male and female patients. **Supplementary fig. 2B.** The M.tb spoligotypes distribution among different age groups.

## Data Availability

The data and materials supporting this article are included within the article.

## Abbreviations

M.tb: *Mycobacterium tuberculosis*
MTBC: *Mycobacterium tuberculosis* bacterial complex
EAI: East African Indian
CAS: Central Asian Strain
LAM: Latin America and Mediterranean
PGRS: Polymorphic GC-rich repetitive sequences
MIRU: Mycobacterial interspersed repetitive unit
SIT: Spoligotype international type
OMR: Ofloxacin mono resistant
DS: Drug sensitive
EPTB: Extra pulmonary tuberculosis
MIC: Minimum inhibitory concentration
IL: Interleukin
CCL: Chemokine (C-C motif) ligand
MCP: Monocyte chemoattractant protein
MIP: Macrophage inflammatory protein
CXCL: Chemokine (C-X-C motif) ligand
MIG: Monokine induced by gamma interferon
IP-10: IFNγ inducible protein 10
ITAC 1: Interferon–inducible T cell alpha chemoattractant 1
