# Supplementary material for: The recent trend in mycobacterial strain diversity among extra pulmonary lymph node tuberculosis and their association with drug resistance and the host immunological response in South India
Source: BMC Infect Dis. 2020 Nov 26;20:894. doi: 10.1186/s12879-020-05597-0 (PMC7690019; doi:10.1186/s12879-020-05597-0)
Supplement: Supplementary file 1 — Supplementary Table 1. Demographic and clinical details of the study population [file 12879_2020_5597_MOESM1_ESM.docx]

**Supplementary Table 1** Demographic and clinical details of the study population

| **ID** | **Smear** | **Culture** | **Octal code** | **SIT No** | **Lineage** |
| --- | --- | --- | --- | --- | --- |
| LM009 | 1+ | 16 Col | 477777777413071 | 11 | EAI3-IND |
| LM012 | Neg | 1+ | 477777777413071 | 11 | EAI3-IND |
| LN014 | Neg | 1+ | 777777777760771 | 53 | T1 |
| LN016 | Pos | 1+ | 000000000003771 | 1 | Beijing |
| LN039 | Neg | 1+ | 474077777413071 | Orphan | Orphan |
| LN041 | Neg | Pos | 777777777413731 | 48 | EAI1-SOM |
| LN043 | Neg | 1+ | 777777777760771 | 53 | T1 |
| LN048 | Neg | 1+ | 777777777760771 | 53 | T1 |
| LN056 | Pos | 2+ | 077767777413731 | Orphan | Orphan |
| LN057 | Neg | Pos | 474347777413771 | Orphan | Orphan |
| LN059 | Neg | pos | 703777740003771 | 26 | CAS1-Delhi |
| LN065 | Neg | 1+ | 775177747413771 | Orphan | Orphan |
| LN066 | Neg | 1+ | 477777777413071 | 11 | EAI3-IND |
| LN067 | Neg | 1+ | 777775607413771 | Orphan | Orphan |
| LN068 | Neg | 1+ | 777777777413731 | 48 | EAI1-SOM |
| LN071 | Neg | 1+ | 477767777413171 | Orphan | Orphan |
| LN072 | Neg | 2 col | 474177777413771 | 938 | EAI5 |
| LN075 | Neg | Pos | 777776777760601 | 137 | X2 |
| LN076 | Neg | 1+ | 777777757413771 | 591 | EAI6-BGD1 |
| LN078 | Pos | Pos | 477777777413071 | 11 | EAI3-IND |
| LN079 | Pos | 1+ | 337777777413731 | 270 | EAI1-SOM |
| LN081 | Neg | 1 col | 777777777760771 | 53 | T1 |
| LN082 | Neg | 1+ | 000000000003771 | 1 | Beijing |
| LN083 | Neg | 1+ | 777777607760771 | 42 | LAM9 |
| LN084 | Neg | 5 col | 777777774000771 | 1952 | Unknown |
| LN085 | Neg | 1+ | 477777777413071 | 11 | EAI3-IND |
| LN088 | Neg | 1+ | 477767677413071 | Orphan | Orphan |
| LN089 | Neg | 1 Col | 777777777760771 | 53 | T1 |
| LN091 | Neg | 16 Col | 777777660020761 | Orphan | Orphan |
| LN093 | Neg | 1+ | 477777777413071 | 11 | EAI3-IND |
| LN094 | Neg | 1+ | 477777777413771 | 126 | EAI5 |
| LN095 | Neg | 11 col | 777777777413731 | 48 | EAI1-SOM |
| LN096 | Neg | 1+ | 477777777413071 | 11 | EAI3-IND |
| LN097 | Neg | 19 col | 477777777413071 | 11 | EAI3-IND |
| LN100 | Neg | 1+ | 474347677412071 | Orphan | Orphan |
| LN103 | Neg | 1+ | 477777777413071 | 11 | EAI3-IND |
| LN104 | Neg | 1+ | 703777740003771 | 26 | CAS1-Delhi |
| LN105 | Neg | 1+ | 703777700003771 | 142 | CAS1-Delhi |
| LN106 | Neg | 1+ | 000000000003771 | 1 | Beijing |
| LN107 | Neg | 1+ | 737777757413771 | 3484 | EAI6-BGD1 |
| LN108 | Neg | 12 col | 474377777413771 | 340 | EAI5 |
| LN109 | Neg | 15 col | 474347777413771 | Orphan | Orphan |
| LN111 | Neg | 1+ | 471777777413071 | 2238 | EAI3-IND |
| LN112 | Neg | 19 Col | 467777777413031 | 474 | EAI5 |
| LN113 | Neg | 11 Col | 477777777413031 | 355 | EAI3-IND |
| LN114 | Neg | 11 Col | 401777777413071 | 473 | EAI3-IND |
| LN116 | Neg | 1+ | 700377400000771 | 601 | CAS |
| LN119 | Neg | 1+ | 706323777740771 | Orphan | Orphan |
| LN120 | Neg | 1+ | 400077717013771 | Orphan | Orphan |
| LN121 | Neg | 1+ | 477777777413071 | 11 | EAI3-IND |
| LN124 | No smear | 1+ | 404323777413771 | Orphan | Orphan |
| LN127 | Neg | 4 Col | 477777777413071 | 11 | EAI3-IND |
| LN135 | Neg | 1+ | 477777777413071 | 11 | EAI3-IND |
| LN136 | Neg | 1+ | 477777777413071 | 11 | EAI3-IND |
| LN137 | Neg | 1+ | 477777777413071 | 11 | EAI3-IND |
| LN138 | 1+ | 1+ | 777777777413711 | 517 | EAI5 |
| LN139 | Neg | 1+ | 477777777413071 | 11 | EAI3-IND |
| LN142 | Neg | 1+ | 474377777413700 | Orphan | Orphan |
| LN145 | Neg | 1+ | 407777374000771 | Orphan | Orphan |
| LN146 | Neg | 1 col | 703777700000371 | 1345 | Unknown |
| LN147 | Neg | 1+ | 401377577413771 | Orphan | Orphan |
| LN149 | Neg | 2+ | 477777777413071 | 11 | EAI3-IND |
| LN152 | 1+ | 1+ | 474177777413771 | 938 | EAI5 |
| LN153 | Neg | 1+ | 477777777413071 | 11 | EAI3-IND |
| LN155 | Neg | 1+ | 404323777413771 | Orphan | Orphan |
| LN158 | Neg | 14 | 777777777740771 | 172 | T1 |
| LN161 | 1+ | 1+ | 703777740003771 | 26 | CAS1-Delhi |
| LN162 | Pos | 2+ | 477777777413771 | 126 | EAI5 |
| LN166 | Neg | 1+ | 477777777413071 | 11 | EAI3-IND |
| LN168 | Neg | 2 col | 477775747413771 | Orphan | Orphan |
| LN171 | 1+ | 3+ | 477777777413071 | 11 | EAI3-IND |
