# Supplementary figures and images for: The recent trend in mycobacterial strain diversity among extra pulmonary lymph node tuberculosis and their association with drug resistance and the host immunological response in South India
Source: BMC Infect Dis. 2020 Nov 26;20:894. doi: 10.1186/s12879-020-05597-0 (PMC7690019; doi:10.1186/s12879-020-05597-0)

Supplementary Figure 1

A

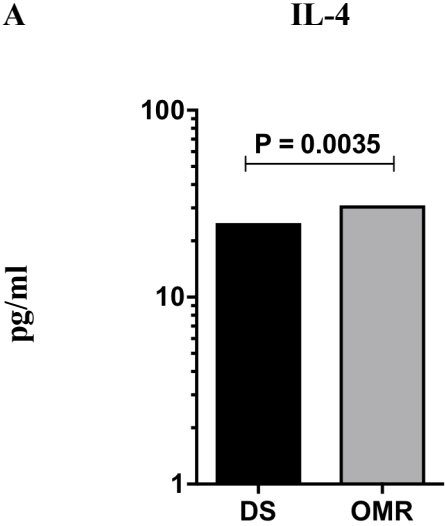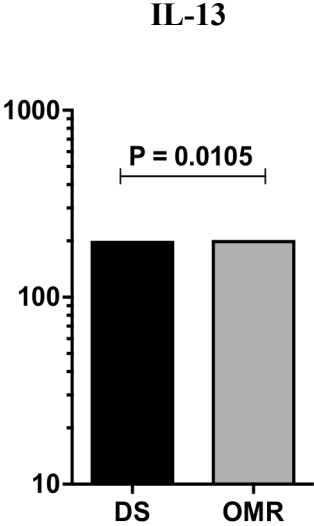

B

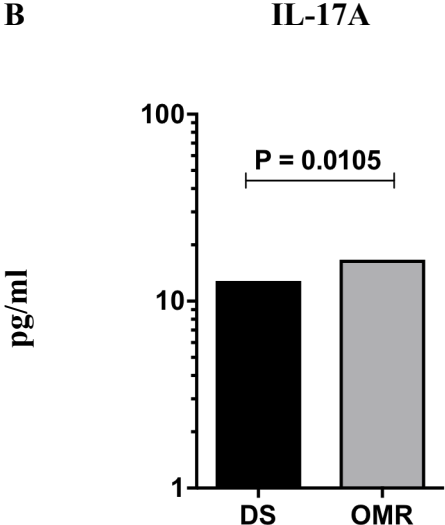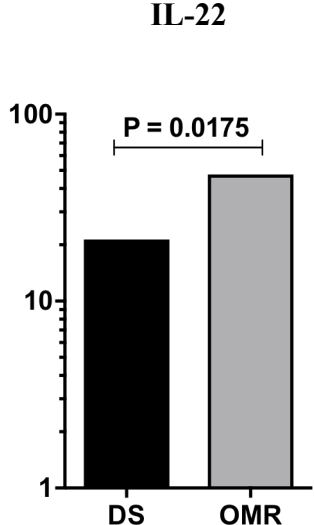

Supplement: Supplementary file 2 — Supplementary Fig. 1. Diminished plasma levels type 2 and type 17 cytokines associated with individuals infected with drug sensitive strain. The systemic levels of IL-4, IL-13, IL-17A and IL-22 were measured by ELISA between the drug sensitive (DS, n = 10) and orphan mono resistant (OMR, n = 3) strain infected individuals. The data were shown as bar graph and P values were calculated using the Mann-Whitney U test. [file 12879_2020_5597_MOESM2_ESM.pdf]

Supplementary Figure 2A

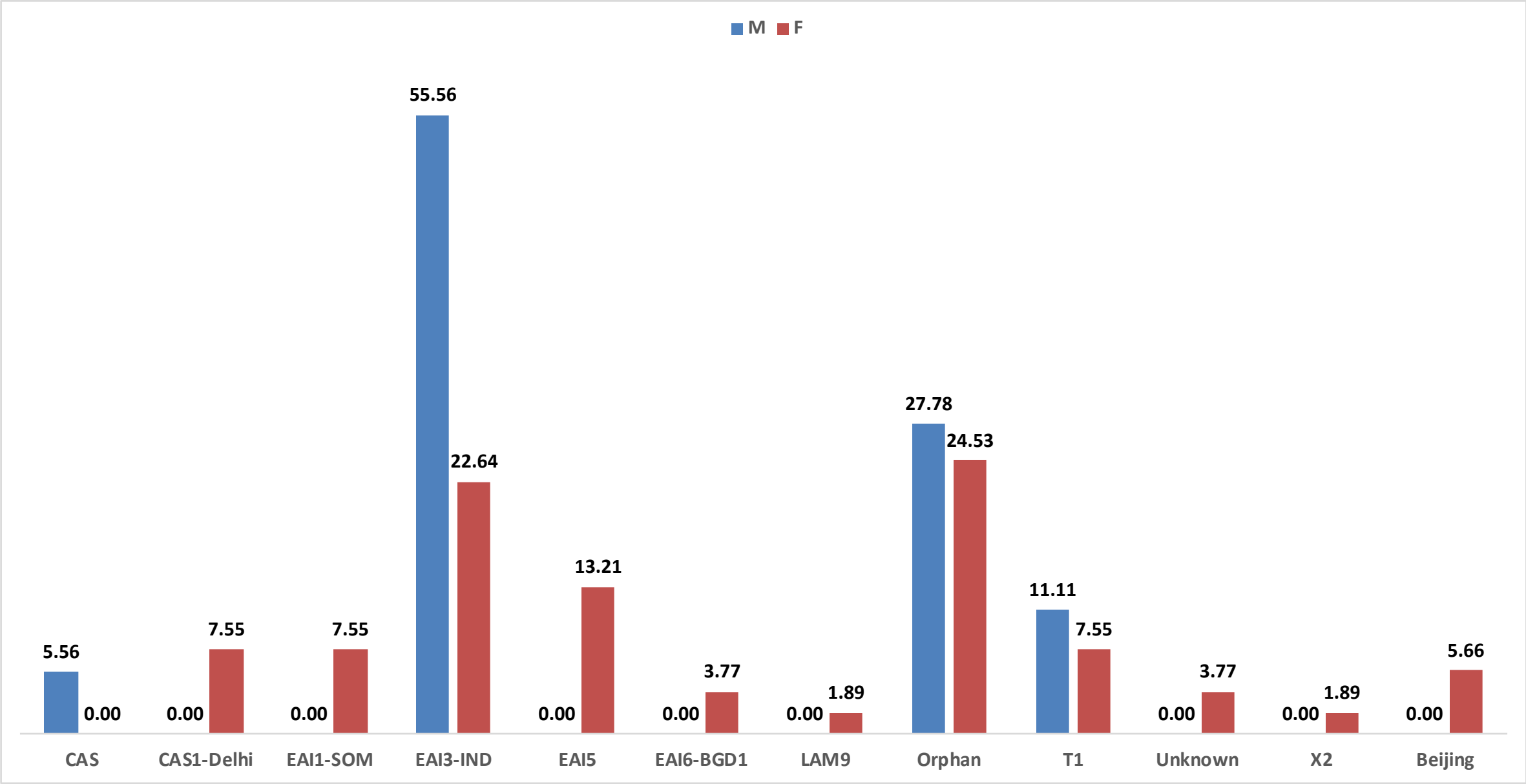

Supplement: Supplementary file 3 — Supplementary fig. 2A. The M.tb spoligotypes distribution among male and female patients. Supplementary fig. 2B. The M.tb spoligotypes distribution among different age groups. [file 12879_2020_5597_MOESM3_ESM.zip › Supp Fig 2AR4.pdf]

Supplementary Figure 2B

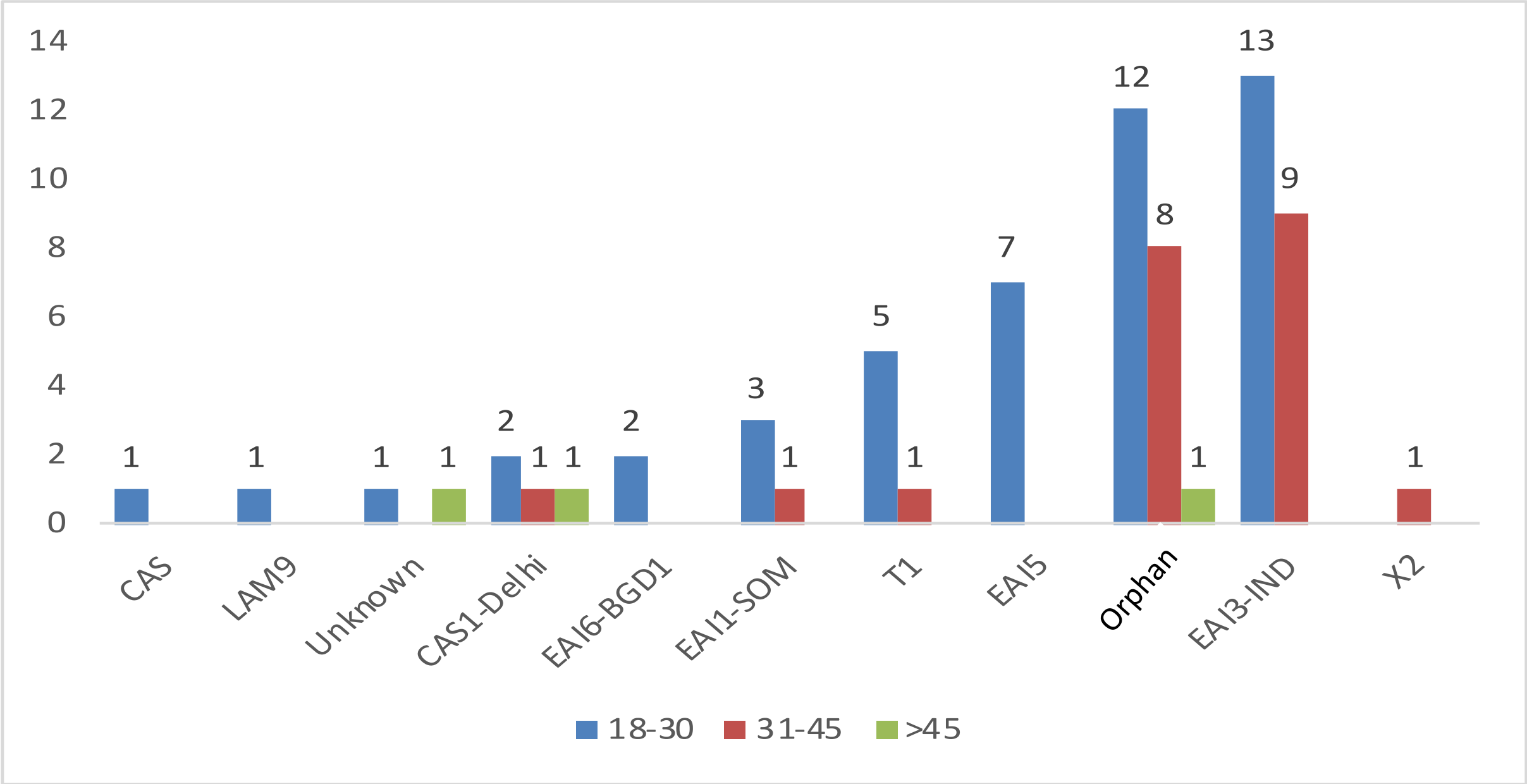

Supplement: Supplementary file 3 — Supplementary fig. 2A. The M.tb spoligotypes distribution among male and female patients. Supplementary fig. 2B. The M.tb spoligotypes distribution among different age groups. [file 12879_2020_5597_MOESM3_ESM.zip › Supp Fig 2BR4.pdf]
